# High Concentrations of L-Ascorbic Acid Specifically Inhibit the Growth of Human Leukemic Cells via Downregulation of *HIF-1α* Transcription

**DOI:** 10.1371/journal.pone.0062717

**Published:** 2013-04-23

**Authors:** Hiroshi Kawada, Mitsuyo Kaneko, Masakazu Sawanobori, Tomoko Uno, Hideyuki Matsuzawa, Yoshihiko Nakamura, Hiromichi Matsushita, Kiyoshi Ando

**Affiliations:** 1 Division of Hematology/Oncology, Department of Medicine, Tokai University School of Medicine, Isehara, Kanagawa, Japan; 2 Research Center for Regenerative Medicine, Tokai University School of Medicine, Isehara, Kanagawa, Japan; 3 Department of Laboratory Medicine, Tokai University School of Medicine, Isehara, Kanagawa, Japan; Rice University, United States of America

## Abstract

We examined the antileukemic effects of high concentrations of L-ascorbic acid (high AA) on human leukemic cells. In vitro, high AA markedly induced apoptosis in various leukemic cell lines by generating hydrogen peroxide (H_2_O_2_) but not in normal hematopoietic stem/progenitor cells. High AA significantly repressed leukemic cell proliferation as well as neoangiogenesis in immunodeficient mice. We then noted that in leukemic cells, *HIF-1α* transcription was strongly suppressed by high AA and correlated with the transcription of *VEGF*. Our data indicate that exposure to high AA markedly increased the intracellular AA content of leukemic cells and inhibited the nuclear translocation of NF-κB, which mediates expression of HIF-1α. We next generated K562 cells that overexpressed *HIF-1α* (K562-HIF1α cells) and assessed the mechanistic relationship between inhibition of *HIF-1α* transcription and the antileukemic effect of high AA. The ability of high AA to induce apoptosis was significantly lower in K562-HIF1α cells than in K562 cells in vitro. We found that expression of HIF-1α-regulated antiapoptotic proteins of the Bcl-2 family, such as Mcl-1, Bcl-x_L_, and Bcl-2, was significantly suppressed by high AA in K562 cells, but was sustained at higher levels in K562-HIF1α cells, regardless of high AA exposure. Moreover, repression of cell proliferation and neoangiogenesis by high AA was completely abrogated in mice receiving transplants of K562-HIF1α cells. These results indicate that, along with H_2_O_2_ generation, downregulation of *HIF-1α* transcription plays a crucial role in growth inhibition of human leukemic cells by high AA.

## Introduction

Pauling and Cameron were the first to report that when L-ascorbic acid (AA) was given intravenously to human cancer patients for 10 days and then orally in pharmacologic doses of 10 g daily, it was effective in treating some cancers and in improving patient survival [1], [2]. The same oral dose had no therapeutic effects on cancer patients in 2 subsequent double-blind placebo-controlled trials [3], [4]. However, we thought that it was important to examine anew the role of AA in cancer treatment for the reasons that follow: (i) the route of AA administration leads to large differences in plasma concentrations, and intravenous administration results in 70-times higher plasma concentration than oral administration [5]; (ii) high concentrations of AA (high AA) administered intravenously exert remarkable anticancer effects by generating hydrogen peroxide (H_2_O_2_) in the extracellular fluid of tumor-bearing animals [6], [7]; and (iii) recent clinical studies also demonstrate the antitumor effects of intravenous high AA in patients with different types of cancers [8], [9]. Further, it is remarkable that the cytotoxic effects of high AA appear to be cancer cell-type specific [7].

In the present study, we attempted, therefore, to determine whether high AA exerts significant cytotoxic effects against human leukemic cells in vitro and in vivo. We confirm here that the leukemic cell-specific cytotoxic effects of high AA were caused by the generation of H_2_O_2_. Further, while HIF-1α plays an important role biologically and clinically in myeloid and lymphoid leukemias [10]–[15], we found that high AA strongly inhibited HIF-1α expression in leukemic cells.

HIF-1 is composed of an inducible (HIF-1α) and a constitutively expressed subunit (HIF-1β) [16]. HIF-1α contains an oxygen-dependent degradation domain, which when hydroxylated by specific prolyl hydroxylases, binds the von Hippel–Lindau protein, leading to the ubiquitination of HIF-1α and its degradation by the 26S proteasome. At low oxygen levels, the prolyl hydroxylases lose their activity, which prevents hydroxylation and subsequent binding to the von Hippel–Lindau protein [17], [18]. This results in HIF-1α stabilization, nuclear translocation, dimerization with the β-subunit, and binding to recognition elements in the promoters of target genes.

AA facilitates the hydroxylation of HIF-1α via the stimulation of the prolyl hydroxylases [19], [20]. However, we have shown here that high AA markedly inhibit the expression of HIF-1α in leukemic cells at the level of transcription. We have further demonstrated that one important mechanism underlying this response is the transcriptional regulation of HIF-1α by the redox-sensitive transcription factor NF-κB, which has been shown to bind at a distinct element in the proximal promoter of *HIF-1α* under not only hypoxic but also non-hypoxic conditions and regulate *HIF-1α* transcription [21]. Most important, the inhibition of HIF-1α expression is considered to play a crucial role in the antileukemic effects of high AA.

## Materials and Methods

### Cells

The human leukemic cell lines, K562 (blast crisis of chronic myeloid leukemia), HL60 (promyelocytic leukemia), MOLM14 (monocytic leukemia), NB4 (promyelocytic leukemia), Jurkat (T-lymphoblastic leukemia), and Raji (B-lymphoblastic leukemia), were maintained in RPMI 1640 medium supplemented with 10% heat-inactivated fetal bovine serum (FCS) and antibiotics (100 U penicillin/ml and 100 μg streptomycin/ml) at 37°C in a humidified 5% CO_2_ atmosphere. The MOLM-14 cell line [22] was kindly provided by the Cell Biology Institute, Research Center, Hayashibara Biochemical Laboratories. The NB4 cell line was purchased from the German Collection of Microorganisms and Cell Cultures. The remaining cell lines were purchased from the American Type Culture Collection. Human umbilical cord blood (CB) samples were harvested from subjects quickly after birth, after written informed consent was obtained in accordance with the Declaration of Helsinki and with approval from the Tokai University Committee on Clinical Investigation. The CD34^+^ cell fraction was prepared using the CD34 Progenitor Cell Isolation Kit (Miltenyi Biotec) [23]. The CB-CD34^+^ cells were frozen in a medium supplemented with dimethylsulfoxide and FCS using a step-down freezing procedure and placed in liquid nitrogen. Aliquots of frozen samples were thawed just before use. The thawed cells were washed twice and viability was determined using trypan blue. When cell viability was more than 95%, the samples were subjected to further studies. To prepare K562 cells that overexpressed HIF-1α, we transfected 293T cells with CSII-HIF1α-IRES-EGFP lentiviral vectors for 72 h and collected the supernatant. K562 cells were incubated with the highly concentrated viral supernatant at a multiplicity of infection of 50 for 24 h. Green fluorescent protein-positive K562 cells were then sorted using a FACSVantage cell sorter (Becton Dickinson).

### AA

AA was buffered to pH 7.0 with sodium hydroxide and prepared immediately before use.

### H_2_O_2_ assay

AA was added to the medium in 96-well culture plates at the concentrations indicated in the figures. H_2_O_2_ was quantitated using a Chemiluminescent H_2_O_2_ Detection Kit (Assay Designs) according to the manufacturer's protocol.

### Cell viability assays

AA was added at varying concentrations to 96-well culture plates containing 5×10^3^ cells/well. Saline solution was used as a vehicle control. One hour later, cells were washed and resuspended in the culture medium. Seventy-two hours later, the viability of the cells was measured with a nonradioactive cell proliferation assay using the Cell Counting Kit-8 (Dojindo) according to the manufacturer's protocol.

### Measurement of apoptosis

Ten thousand cells were incubated with the vehicle, 2800 μM AA, or 2800 μM AA and 600 U/ml of catalase for 1 h and then washed and cultured in the medium. The cells were harvested 18 h later and stained with fluorescein isothiocyanate (FITC)- or allophycocyanin (APC)-labeled annexin V (BD Biosciences) and propidium iodide (PI) (Roche), or with primary anti-cleaved caspase-3 antibody (Cell Signaling) and secondary phycoerythrin (PE)-labeled antibody (eBioscience), according to the manufacturer's instructions. The treated cells were then analyzed using a FACScan flow cytometer (Becton Dickinson).

### Measurement of intracellular catalase activity

The intracellular catalase activity of 2×10^4^ cells was measured using a Fluorescent Catalase Detection Kit (Fluoro: Catalase^TM^, Cell Technology) according to the manufacturer's instructions. Briefly, the kit utilizes a non-fluorescent substrate, 10-acetyl-3,7-dihydroxyphenoxazine, which is converted by residual H_2_O_2_ to the fluorescent molecule resorufin.

### Histochemical catalase assay

Cell preparations (2×10^5^) were placed on glass slides in a cytospin centrifuge and fixed with 4% paraformaldehyde (PFA) for 5 min. The slides were incubated with a rabbit anti-catalase antibody (Sigma Aldrich) overnight at 4°C and then with a horseradish peroxidase-conjugated anti-rabbit antibody (GE Healthcare, Japan) for 4 h at 4°C. After the slides were washed, they were stained with 3,3′-diaminobenzidine tetrahydrochloride, and images were acquired using a digital camera (AxioCam MRc5, Carl Zeiss).

### Xenograft and Treatment Procedures

Leukemic cells (2×10^6^) were mixed with 100 μl basement membrane matrix (BD Biosciences), and the mixture was transplanted subcutaneously into the right flank of 8-week-old nude mice. On day 8 after transplantation, the tumor volume was measured, and the mice were then injected intravenously with 100 μl of AA at a high concentration (0.5 mg/g body weight, which is similar to the pharmacologic doses for humans and rats [6], [7], [24]–[26]), or saline solution as a bolus twice daily for the designated periods. The tumor volume was measured at the times indicated in the figures. All experimental procedures and protocols involving animals were approved by the Animal Care Committee of Tokai University and were in compliance with the ARRIVE guidelines [27].

### Immunohistochemical and immunocytochemical analyses

Isoflurane inhalation was used to anesthetize mice, and the tumor was perfused from the apex of the heart with phosphate-buffered saline (PBS) and fixed by perfusion with 4% PFA in PBS. The tumor was then dissected and immersed in 4% PFA overnight at 4°C, embedded in O.C.T. compound (Sakura Finetek, Japan), and then frozen in liquid nitrogen. Cryostat sections (6 μm thick) of the tumor or cytospin specimens of leukemic cells fixed with 4% PFA were stained with specific antibodies and incubated overnight at 4°C. Rat anti-mouse CD31 (BD Sciences) and rabbit anti-NF-κB p65 antibodies (Cell Signaling Technology) were used as primary antibodies. The slides were then incubated with a secondary antibody conjugated with Alexa488 (Life Technologies). Nuclei were stained with 4′,6-diamidino-2-phenylindole (DAPI; Life Technologies). The slides were observed using a confocal laser-scanning microscope (LSM510 META spectrometer, Carl Zeiss).

### Analysis of angiogenesis-related and antiapoptotic molecules

Cells were treated with a high AA (2800 μM). After 1 h, the cells were washed, cultured for 24 h, unless otherwise indicated, and then assayed.

### Quantitative real-time polymerase chain reaction

RNA was isolated using the RNeasy Micro Kit (QIAGEN) and reverse transcribed. Each target cDNA was polymerase chain reaction (PCR)-amplified on the same plate by using the TaqMan(R) Gene Expression Assays (Life Technologies Corporation) and the ABI 7300 Real-Time PCR System (Applied Biosystems). The PCR primers used were derived from *HIF-1α* (Applied Biosystems, Assay ID; Hs00936376_m1) and *VEGF* (Applied Biosystems, Assay ID; Hs00900055_m1). The relative amounts of target genes were determined in reference to 18S rRNA. Comparative threshold cycle (C_T_) analysis was used to quantify transcripts. The value was calculated by the expression 2^−ΔΔCT^.

### Western blotting

Cells were harvested and washed, and the pellets were suspended in 0.1 ml of ice-cold TNE buffer and incubated on ice for 10 min. When subcellular fractions were prepared, the Subcellular Proteome Extraction Kit (Calbiochem) was used according to the manufacturer's instructions. The lysates were then centrifuged, and the supernatants were boiled in SDS sample buffer. The proteins were separated on SDS-polyacrylamide gels, electroblotted onto a nitrocellulose membrane, and detected using the ECL Plus Western blotting analysis system (GE Lifesciences) using specific antibodies. Anti-HIF-1α and anti-β-actin antibodies were purchased from BD Biosciences and Sigma-Aldrich, respectively. Anti-p-IκB, anti-NF-κB, anti-Bcl-2, anti-Bcl-x_L_, anti-caspase-3, and anti-lamin A/C antibodies were purchased from Cell Signaling Technology. Anti-Mcl-1, anti-Sp1, anti-Sp3, and anti-Sp4 were purchased from Santa Cruz Biotechnology, Inc.

### Quantitative assays for intracellular AA content

Cells were treated with 2800 μM AA for 1 h, washed twice in PBS, and then assayed for AA content using a vitamin C assay kit (Shima Laboratories) according to the manufacturer's instructions. Briefly, AA in a given sample is converted by the oxidizing agent to dehydroascorbic acid. Dehydroascorbic acid is then derivatized with 2,4-dinitrophenylhydrazine. Total vitamin C (AA + dehydroascorbic acid) concentration is determined by the specific ultraviolet light (UV) absorption of the 2,4-dinitrophenylhydrazine derivative.

### Statistics

All the experimental results have been expressed as the arithmetic mean and standard deviation (SD) values. Student's *t*-test was used to evaluate the statistical significance of the differences between unpaired groups.

## Results

### Cancer-specific cytotoxic effect of high AA on human hematopoietic cells

**Figure 1:**
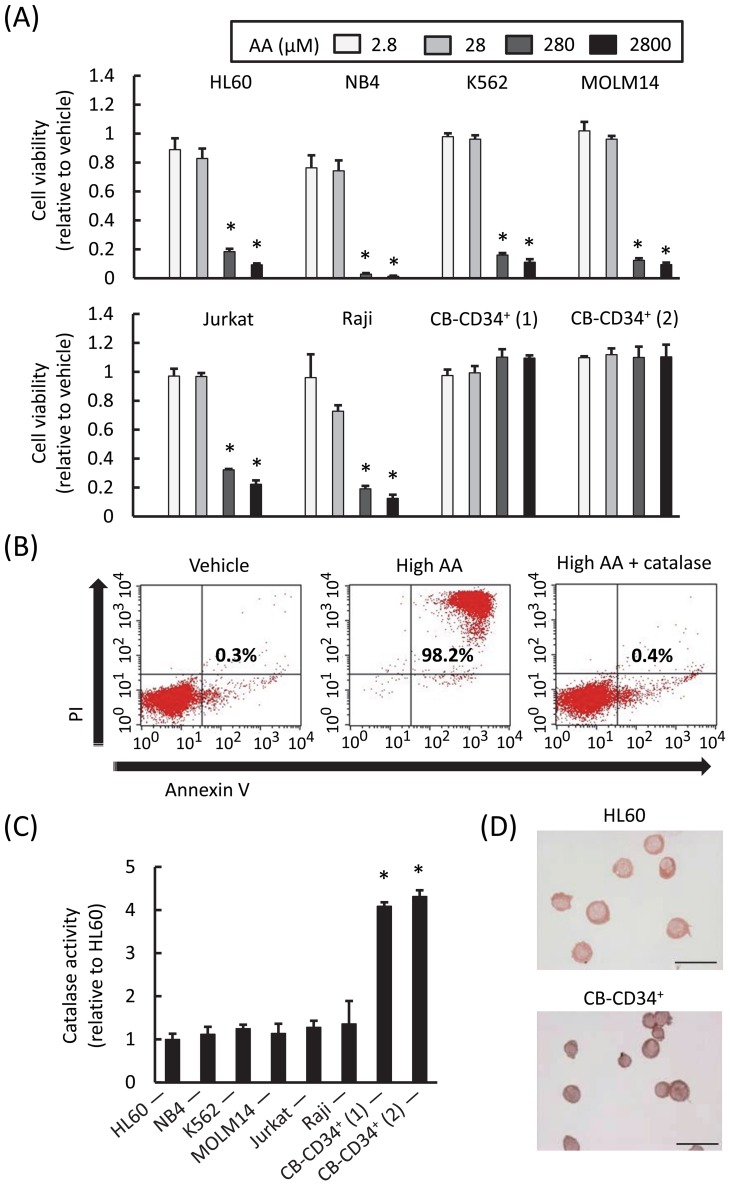
In vitro effects of AA on human leukemic and CB-CD34^+^ cells, relative to catalase activity. A) Cell viability assay of various leukemic cell lines and 2 independent isolates of CB-CD34^+^ cells. Cells were treated with different concentrations of AA for 1 h, and then washed, cultured, and analyzed after 72 h. The viability of all cell lines reduced significantly in the presence of 280 and 2800 µM AA (*P<0.0001, as compared with vehicle), but this finding was not obtained for CB-CD34^+^ cells (*P*>0.05). The values represent the mean ± SD values of quadruplicate samples. B) Flow cytometric measurement of apoptosis of HL60 cells. Cells were treated with vehicle or AA for 1 h, and then washed, cultured, and analyzed after 18 h. Representative profiles are shown. The annexin V^+^ propidium iodide (PI)^+^ cell fraction indicates apoptotic cells. Note that AA-induced apoptosis was almost completely abrogated by the addition of catalase. C) Intracellular catalase activity. Leukemic cells generally expressed lower catalase activities than did CB-CD34^+^ isolates (*P<0.001, as compared with each cell line). The values represent the mean ± SD values of quadruplicate samples. D) Histochemical analysis demonstrated lower catalase activity in HL60 cells than in CB-CD34^+^ cells. The bars indicate 50 μm.

We first assessed the effect of high AA on the viability of various human leukemic cell lines as well as on normal hematopoietic stem/progenitor cells in vitro. Addition of 280 and 2800 μM of AA, which are approximately 6 and 60-times higher than the physiological level, produced significant amounts of H_2_O_2_ after 1 h incubation (34.4±4.1 and 134.0±11.8 μM, respectively) and reduced the viability of all myeloid and lymphoid leukemic cells tested but not that of CB-CD34^+^ cells (Figure 1A). We further found that high AA induced apoptosis in leukemic cells and that this effect was almost completely abrogated by the addition of catalase (Figure 1B). It is important to note that the leukemic cell lines tested generally possessed lower catalase activities than did normal CB-CD34^+^ cells (Figures 1C and 1D). Thus, we conclude that the induction of apoptosis by high AA was due to the generation of H_2_O_2_ and was specifically observed in leukemic cells that expressed relatively lower catalase activities.

### Inhibitory effect of high AA on leukemic progression in vivo

**Figure 2:**
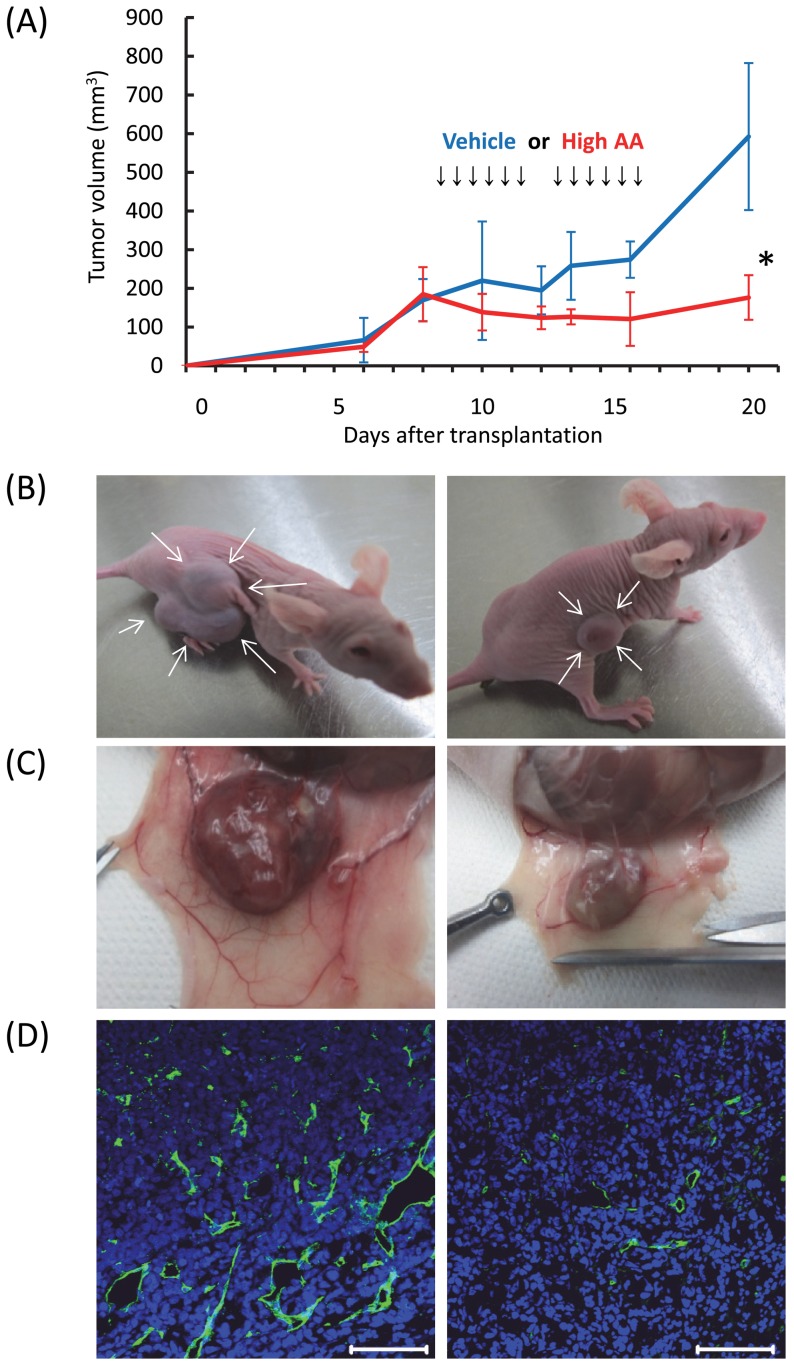
In vivo effects of high AA on progression of leukemia. A) High AA or the vehicle was injected intravenously for 6 days with a rest period of 2 days between 3 daily injections of mice transplanted with HL60 cells. Compared with vehicle (blue line), high AA (red line) significantly inhibited tumor growth (*P<0.01). The values represent the mean ± SD values of 5 mice. B) Appearance of mice treated with vehicle (left) and high AA (right), 4 days after the final injection. C) Representative macroscopic appearance of tumors of mice treated with the vehicle (left) and high AA (right). Note that the tumors of high AA-treated mice were smaller and less erythematous than those of vehicle-treated mice. D) Immunohistochemical analysis of tumor neoangiogenesis in mice treated with the vehicle (left) and high AA (right). The green and blue signals represent CD31 and 4′,6-diamidino-2-phenylindole (DAPI), respectively. The bars indicate 100 μm.

We next examined the effect of high AA on the progression of leukemia by using an experimental transplantation model. We mixed HL60 cells and basement membrane matrix (BD Biosciences), transplanted the mixture subcutaneously into the right flank of nude mice, and injected high AA or vehicle intravenously. This procedure enabled a precise assessment of tumor burden over time. There were significant differences in tumor volumes between vehicle- and high AA-treated mice 4 days after the final injection (Figures 2A and 2B). We then killed the mice and found that tumor neoangiogenesis was less evident in high AA-treated mice than in vehicle-treated mice (Figures 2C and 2D).

### Inhibitory effect of high AA on HIF-1α expression in leukemic cells

**Figure 3:**
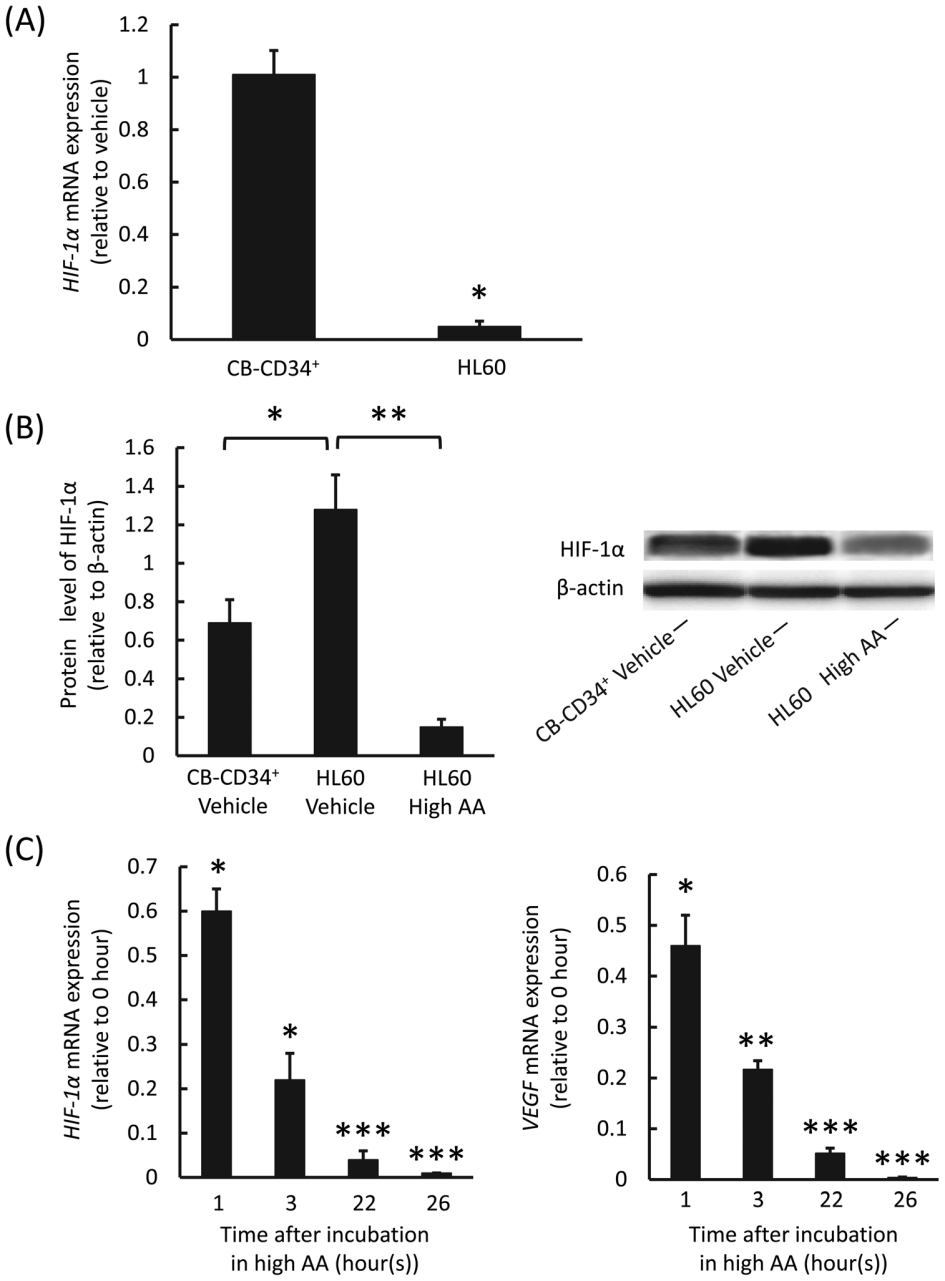
Expression of angiogenesis-related molecules in human leukemic and CB-CD34^+^ cells exposed to the vehicle or to high AA. A) Quantitative real-time PCR (qRT-PCR) analysis of *HIF-1α* mRNA in CB-CD34^+^ and HL60 cells. The cells were treated with vehicle or high AA for 1 h, and then washed, cultured, and analyzed after 24 h. There were no significant differences in the expression levels for the 2 conditions (*P*>0.05) in CB-CD34^+^ cells. In contrast, there were significant differences in the expression levels between the 2 conditions (*P<0.0001) in HL60 cells. The values represent the mean ± SD values of triplicate samples. B) Western blotting analysis of HIF-1α in CB-CD34^+^ and HL60 cells. The cells were treated with vehicle or high AA for 1 h, and then washed, cultured, and analyzed after 24 h. There were significant differences in the expression levels (*P<0.01, **P<0.0005). The values are mean ± SD values of triplicate samples. C) Sequential analysis of qRT-PCR results of *HIF-1α* and *VEGF* mRNA in HL60 cells. The cells were treated with high AA for 1 h, and then washed, cultured, and analyzed after 1, 3, 22, and 26 h. The expression of *VEGF* mRNA reduced along with that of *HIF-1α* over time. Compared with the expression levels at 0 h, there were significant differences in the expression levels (*P<0.01, **P<0.001, ***P<0.0001). The values represent the mean ± SD values of triplicate samples.

We next determined the expression of angiogenesis-related molecules in CB-CD34^+^ and leukemic cells in the presence of vehicle or high AA. In CB-CD34^+^ cells, there was no statistically significant difference in the expression of *HIF-1α* mRNA for the 2 conditions (Figure 3A). In contrast, in HL60 cells, expression of *HIF-1α* mRNA markedly decreased because of high AA (Figure 3A). The expression of HIF-1α in HL60 cells was significantly higher than that in CB-CD34^+^ cells in the absence of high AA but markedly reduced in the presence of high AA (Figure 3B). Moreover, mRNA expression of *VEGF*, an HIF-1α-regulated gene, also reduced along with that of *HIF-1α* over time after incubation of HL60 cells with high AA (Figure 3C).

**Figure 4:**
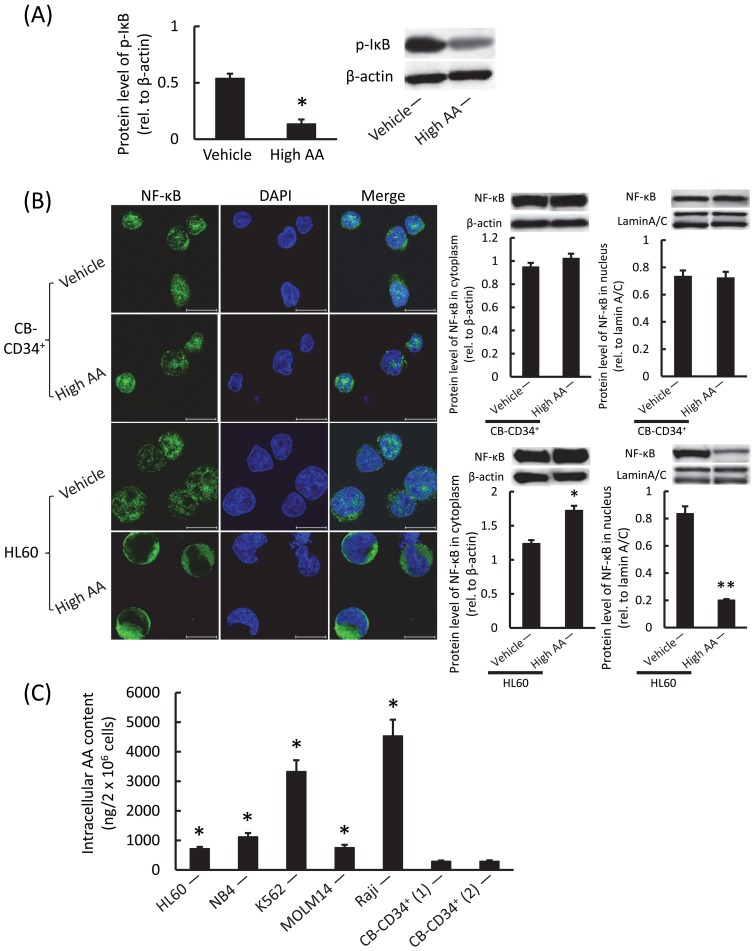
Differences in NF-κB activation and intracellular AA content between human leukemic and CB-CD34^+^ cells in the presence of high AA. A) Western blotting analysis of p-IκB in HL60 cells. Cells were treated with the vehicle or with high AA for 1 h, and then washed, cultured, and analyzed after 24 h. There was a significant difference in the expression levels (*P<0.001). Values represent the mean ± SD of triplicate samples. B) Immunocytochemical (left) and Western blotting (right) analyses of NF-κB in CB-CD34^+^ and HL60 cells. Cells were treated with vehicle or high AA for 1 h, then washed, cultured, and analyzed after 24 h. Note that translocation of NF-κB into the nucleus was markedly decreased in high AA-treated HL60 cells. Green and blue signals represent NF-κB and DAPI, respectively. Bars indicate 20 μm. There were significant differences in the expression levels (*P<0.001, **P<0.0001). The values represent the mean ± SD values of triplicate samples. C) Intracellular AA content of human leukemic cells and 2 different isolates of CB-CD34^+^ cells. Cells were treated with high AA for 1 h, washed in PBS, and analyzed immediately. There were significant differences in the content between leukemic and CB-CD34^+^ cells. *P<0.001, as compared with CB-CD34^+^ cells (1) or (2). The values are mean ± SD values of triplicate samples.

We then attempted to determine how *HIF-1α* mRNA expression was inhibited by high AA in leukemic cells. *HIF-1α* is known to be transcriptionally regulated by NF-κB, and AA inhibits phosphorylation of the NF-κB inhibitor (IκB) [28]. Therefore, we tested for the presence of phosphorylated IκB (p-IκB) and found that the p-IκB level in HL60 cells was significantly reduced by the addition of high AA (Figure 4A). These data indicate that high AA markedly inhibited the translocation of NF-κB into the nucleus of HL60 cells, but not CB-CD34^+^ cells (Figure 4B). We demonstrated further that the intracellular content of AA was much higher in leukemic cells than in normal CB-CD34^+^ cells after incubation with high AA (Figure 4C). These results suggest that the differences in the intracellular uptake of AA reflected the differences seen between high AA-treated human leukemic cells and CD34^+^ cells derived from normal CB in the presence of NF-κB translocation and following HIF-1α expression.

### Relationship between the inhibitory effects of high AA on HIF-1α expression and leukemic progression

**Figure 5:**
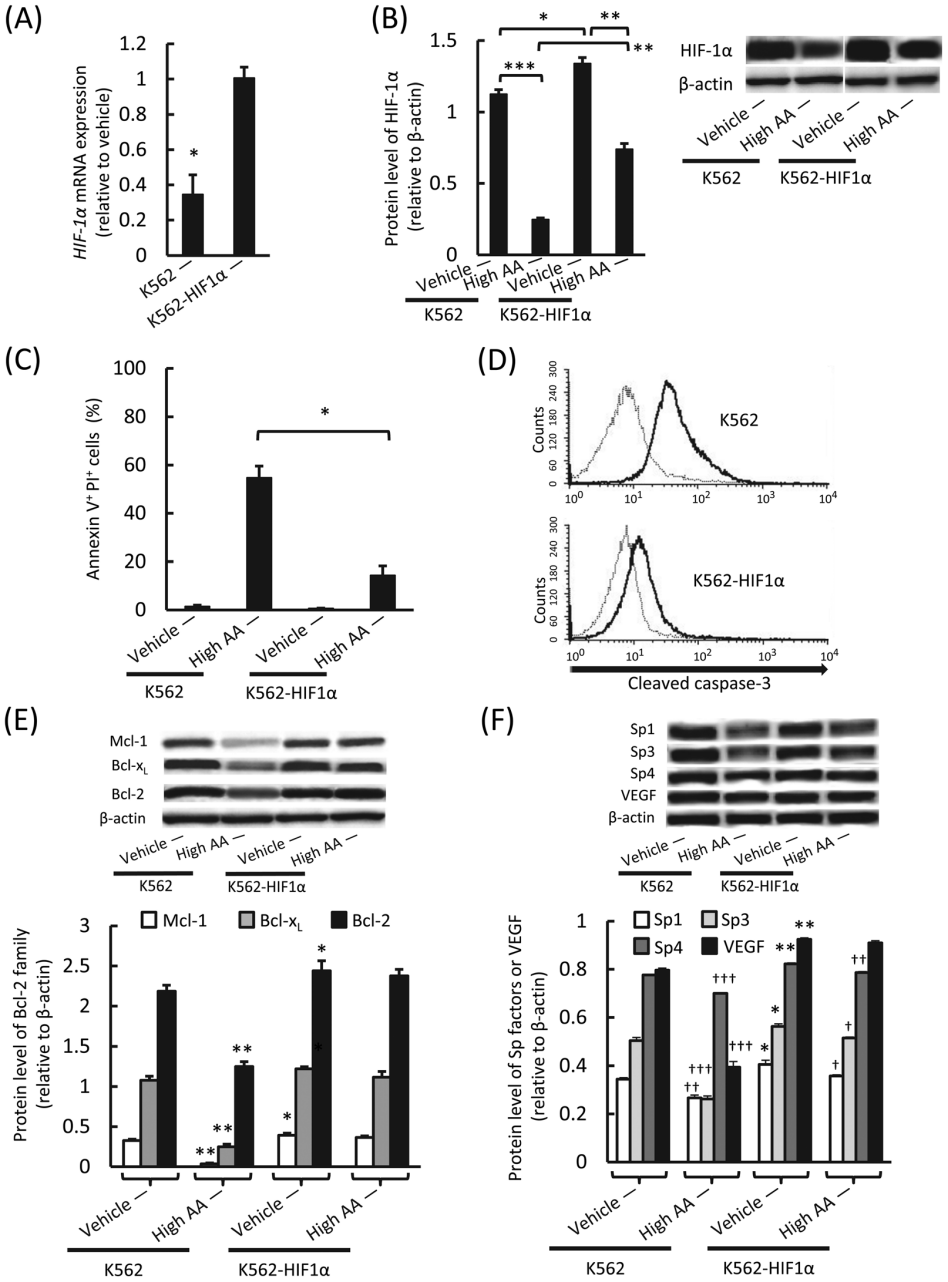
Relationship between antileukemic effects of high AA and HIF-1α expression. A) Quantitative real-time PCR analysis of *HIF-1α* mRNA expression in K562 and K562-HIF1α cells. Cells were treated with the vehicle or high AA for 1 h, washed, cultured in the medium, and analyzed after 24 h. After high AA exposure, *HIF-1α* mRNA expression significantly reduced in K562 (*P<0.01), but not in K562-HIF1α cells (*P*>0.05). The values represent the mean ± SD values of triplicate samples. B) Western blotting analysis of HIF-1α in K562 and K562-HIF1α cells. Cells were treated with vehicle or high AA for 1 h, washed, cultured in the medium, and analyzed after 24 h. High AA exposure significantly reduced the HIF-1α protein level in both types of cells. However, the HIF-1α protein level in K562-HIF1α cells was significantly higher than that in K562 cells after vehicle or high AA exposure. *P<0.01, **P<0.0001, ***P<0.00001. The values represent the mean ± SD values of triplicate samples. C) Flow cytometric measurement of apoptosis of K562 and K562-HIF1α cells. Cells were treated with vehicle or high AA for 1 h, washed, cultured in the medium, and analyzed after 18 h. There was a significant difference in the number of apoptotic (annexin V^+^ propidium iodide (PI)^+^) cells between high AA-treated K562 and K562-HIF1α cells (*P<0. 001). The values represent the mean ± SD values of triplicate samples. D) Flow cytometric measurement of cleaved caspase-3 expressed by K562 and K562-HIF1α cells. Cells were treated with vehicle (gray lines) or high AA (black lines) for 1 h, washed, cultured, and analyzed after 24 h. Activation of caspase-3 by high AA was lower in K562-HIF1α than in K562 cells. E) Western blotting analysis of Mcl-1, Bcl-x_L_, and Bcl-2 in K562 and K562-HIF1α cells. Cells were treated with vehicle or high AA for 1 h, washed, cultured, and analyzed after 24 h. There were significant differences in the expression levels between the vehicle-treated K562 and K562-HIF1α cells (*P<0.05) and between the vehicle-treated and high AA-treated K562 cells (**P<0.0001). There was no significant difference between the vehicle-treated and high AA-treated K562-HIF1α cells (*P*>0.05). The values represent the mean ± SD values of triplicate samples. F) Western blotting analysis of Sp1, Sp3, Sp4, and VEGF. Cells were treated with vehicle or high AA for 1 h, washed, cultured, and analyzed after 24 h. There were significant differences in the expression levels of these molecules between the vehicle-treated K562 and K562-HIF1α cells (*P<0.01, ** *P*<0.0001). There were significant differences in the expression levels of Sp1, Sp3, and Sp4 between the vehicle-treated and high AA-treated K562 or K562-HIF1α cells (^†^ *P*<0.01, ^††^ *P*<0.001, ^†††^ *P*<0.0001). There was a significant difference in the expression level of VEGF between the vehicle-treated and high AA-treated K562 (^†††^ *P*<0.0001), but not between the vehicle-treated and high AA-treated K562-HIF1α cells (*P*>0.05).

Next, we assessed the implications of the inhibition of HIF-1α expression by high AA on leukemic progression by generating *HIF-1α*-overexpressing K562 cells (K562-HIF1α) by using a lentiviral vector. High AA exposure significantly reduced the expression of *HIF-1α* mRNA in K562 but not in K562-HIF1α cells (Figure 5A). The level of HIF-1α in K562-HIF1α cells was also significantly higher than that in K562 cells after vehicle or high AA exposure (Figure 5B). We also found that the induction of apoptosis by high AA was significantly lower in K562-HIF1α than in K562 cells (Figure 5C and 5D). Therefore, we assessed the expression of antiapoptotic proteins of the Bcl-2 family (Mcl-1, Bcl-x_L_, and Bcl-2) because their expression is regulated by HIF-1α in nonmalignant and malignant cells. Moreover, they play a key role in preventing apoptosis mediated by reactive oxygen species (ROS) [14], [29]–[35]. We demonstrated that expression of Mcl-1, Bcl-x_L_, and Bcl-2 was significantly inhibited by high AA in K562 cells but was sustained at a higher level in K562-HIF1α cells, regardless of high AA exposure (Figure 5E). We further assessed the involvement of the pro-oncogenic specificity protein (Sp) transcription factors Sp1, Sp3, and Sp4 in the antileukemic effect of high AA because high AA exhibits anticancer activity towards colon cancer cells. This is due in part to downregulation of Sp transcription factors and Sp-regulated genes, such as *VEGF* [36]. There were significant differences in the expression levels of these molecules between the vehicle-treated K562 and K562-HIF1α cells (Figure 5F). In K562 cells, the expression of Sp1, Sp3, and Sp4 as well as that of VEGF was reduced by high AA (Figure 5F). In K562-HIF-1α cells, the expression of Sp1, Sp3, and Sp4 was reduced by high AA, but the expression of VEGF was not (Figure 5F).

**Figure 6:**
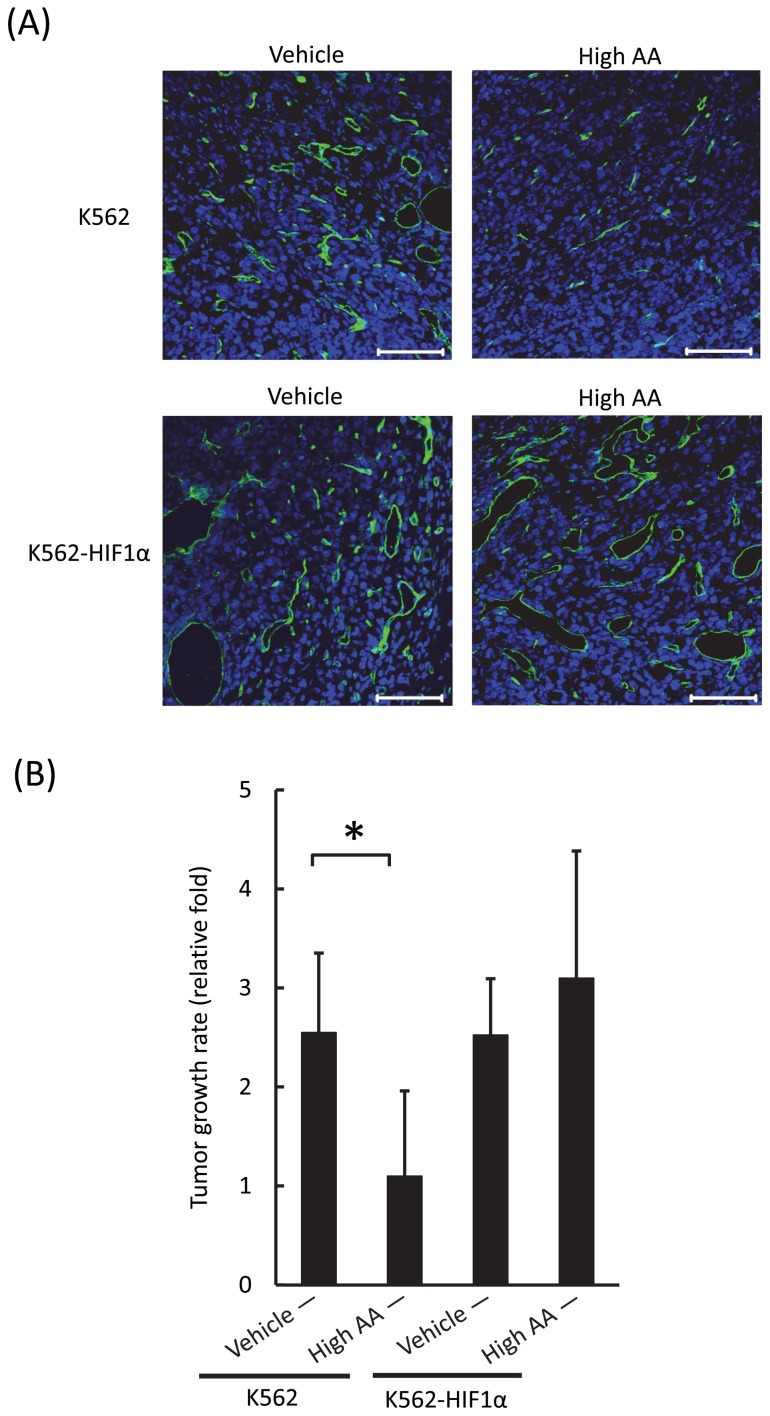
Effects of high AA on tumor growth in the presence or absence of overexpression of ***HIF-1α*** **.** A) Immunohistochemical analysis of tumor neoangiogenesis in vehicle-treated (left) and high AA-treated (right) mice transplanted with K562 (upper column) or K562-HIF1α cells (lower column). The green and blue signals represent CD31 and DAPI, respectively. The bars indicate 100 μm. Note that administration of high AA suppressed tumor neoangiogenesis in mice transplanted with K562 cells, but not in mice transplanted with K562-HIF1α cells. B) In the xenogeneic transplant model, high AA or vehicle was injected for 5 days. Administration of high AA significantly inhibited tumor growth of K562 cells (*P<0.05) but not of K562-HIF1α cells (*P*>0.05). Tumor growth rate was estimated using the following equation: tumor volume on day 4 after high AA treatment/tumor volume just before high AA treatment. The values represent the mean ± SD values for 4 mice.

Finally, we mixed K562 or K562-HIF1α cells in basement membrane matrix, transplanted the mixture into mice, and injected the mice intravenously with the vehicle or high AA. We found that administration of high AA repressed tumor neoangiogenesis only in mice transplanted with K562 cells (Figure 6A). Further, administration of high AA significantly repressed the growth of K562 tumors but did not detectably inhibit the growth of K562-HIF1α tumors in mice (Figure 6B).

## Discussion

AA plays a key role in protecting cells against oxidative damage. Paradoxically, in the presence of Fe^3+^ or Cu^2+^, AA treatment generates ROS, such as H_2_O_2_ [37], and induces apoptosis or necrosis in various malignant cells but not in nonmalignant cells [38]. In the present study, we further investigated these findings and confirmed them using human leukemic and normal hematopoietic cells. We found that high AA induces apoptosis only in the leukemic cells, which we concluded reflects the increasing generation of H_2_O_2_ and relatively low catalase activities [24], [39].

We also found that intravenous administration of high AA repressed proliferation of leukemic cells injected into nude mice. Although high AA are usually given by drip infusion in clinical settings [7], [9], [40], [41], we injected mice with high AA in the form of a bolus, which might have weakened the effect of treatment because of more rapid clearance of AA than by drip infusion [42]. However, we observed a significant antileukemic effect of high AA in the present study. Further, the tumors showed markedly reduced neoangiogenesis. Our present findings demonstrate that high AA strongly inhibits expression of HIF-1α and one of HIF-1α-regulated molecules, VEGF, in leukemic cells. HIF-1α and VEGF are considered as potential targets for cancer therapy because they play an important role in the progression of many types of cancer, including leukemia, and are associated with resistance to therapy and poor prognosis [10]–[13], [43]–[45]. Wang et al. demonstrated that HIF-1α signaling is selectively activated in human leukemic cells even under normoxic conditions [10]. AA facilitates the hydroxylation of HIF-1α via the stimulation of the Fe-dependent hydroxylases that mark this protein for polyubiquitination and subsequent proteosomal degradation [19], [20]. Moreover, Knowles et al. reported that AA reduces HIF-1α protein levels in several human non-hematopoietic cancer cells under normoxic conditions [46]. We have shown here that high AA markedly inhibits the expression of HIF-1α at the level of transcription in leukemic cells.

We have also shown here that in the leukemic cells, high AA inhibited *HIF-1α* transcription by blocking transcriptional activation of NF-κB, which is also constitutively activated in many types of leukemia and is associated with leukemic progression [47]–[49]. Because the leukemic cells used in this study generally possessed significantly higher intracellular levels of AA than normal hematopoietic cells after incubation with high AA, we speculate that while H_2_O_2_ acts to activate NF-κB by increasing phosphorylation of IκB and *HIF-1α* expression [15], [28], AA overcomes the effect of H_2_O_2_ on the regulation of NF-κB activation in the leukemic cells. Further, we conclude that the increased uptake of AA by leukemic cells, also observed by other investigators and possibly associated with an abnormality in AA transport [50]–, reflects the difference in HIF-1α expression levels between leukemic and normal CB-CD34^+^ cells after high AA exposure. The levels of intracellular AA did not closely correlate with the cytotoxic effects of high AA, as shown in Figures 1A and 4C, because the effects were caused largely by the H_2_O_2_ that was generated extracellularly by high AA (Figure 1B).

However, we found that *HIF-1α* overexpression completely abrogated the inhibitory effects of high AA on tumor growth and neoangiogenesis in vivo and significantly diminished the induction of apoptosis by high AA in the leukemic cells in vitro. HIF-1α regulates the expression of Bcl-2 family members such as Mcl-1, Bcl-x_L_, and Bcl-2, which are essential for the growth and survival of leukemic cells because they prevent the induction of apoptosis by ROS [14], [29], [53]–[57]. Here, we demonstrated that high AA significantly suppressed expression of Mcl-1, Bcl-x_L_, and Bcl-2, and induced apoptosis in K562 cells but not in K562 cells that overexpressed *HIF-1α*.

We further assessed the involvement of Sp1, Sp3, and Sp4 in the antileukemic effect of high AA because high AA exhibits anticancer activity towards colon cancer cells, which is due in part to downregulation of Sp transcription factors and Sp-regulated genes [58]. Similar results have been observed in bladder and pancreatic cancer cells treated with H_2_O_2_ or other ROS inducers [59]–[61]. Further, it has been reported that knockdown or downregulation of Sp1, Sp3, and Sp4 represses expression of Sp-regulated genes, including *VEGF* and *BCL-2*, inhibits cancer cell growth, and induces apoptosis [59]–[61]. In the present study, the expression levels of Sp1, Sp3, and Sp4 in K562-HIF1α cells were higher than those in K562 cells, suggesting some interaction between HIF-1α and Sp proteins. Further, the expression of Sp1, Sp3, and Sp4 was also downregulated by high AA in K562 cells, as was observed in colon cancer cells [58]. However, in K562-HIF1α cells, the expression of these factors was also downregulated by high AA, but the expression of VEGF and Bcl-2 was not. These results strongly suggest that marked inhibition of *HIF-1α* transcription and expression of HIF-1α-regulated molecules play a crucial role in the antileukemic effects of high AA along with the generation of H_2_O_2_. However, high AA do not specifically affect the transcription of *HIF-1α* because high AA block the activation of NF-κB, which acts as a transcription factor to regulate the expression of genes involved in the response of leukemic cells to extracellular signals such as *HIF-1α* [48], [49]. Therefore, other molecular mechanisms might also play a role in the response to high AA treatment.

Because the use of high AA appears to be remarkably safe in clinical settings [40], it may provide an alternative option for cancer therapy. However, the anticancer effects of high AA vary among cancers or patients [7], [9], [41]. It is known that an increased number of leukemic cells, normal erythrocytes, or fibroblasts around leukemic cells inversely correlates with high AA-induced leukemic cell death because of increased catalase activity [62]. Therefore, the volume and localization of cancer cells should be considered to obtain more stable clinical effects of high AA. We think that it is reasonable to conclude that combinations with other drugs that compensate for H_2_O_2_ decomposition may also provide a new strategy for eliminating cancer cells [63].
